# Climatic anomaly affects the immune competence of California sea lions

**DOI:** 10.1371/journal.pone.0179359

**Published:** 2017-06-28

**Authors:** Marina Banuet-Martínez, Wendy Espinosa-de Aquino, Fernando R. Elorriaga-Verplancken, Adriana Flores-Morán, Olga P. García, Mariela Camacho, Karina Acevedo-Whitehouse

**Affiliations:** 1 Unit for Basic and Applied Microbiology, School of Natural Sciences, Autonomous University of Queretaro, Queretaro, Mexico; 2 Department of Fisheries and Marine Biology, Centro Interdisciplinario de Ciencias Marinas, Instituto Politécnico Nacional (CICIMAR-IPN), La Paz, Mexico; 3 Department of Human Nutrition, School of Natural Sciences, Autonomous University of Queretaro, Queretaro, Mexico; 4 The Marine Mammal Center, Sausalito, California, United States of America; Midwestern University, UNITED STATES

## Abstract

The past decades have been characterized by a growing number of climatic anomalies. As these anomalies tend to occur suddenly and unexpectedly, it is often difficult to procure empirical evidence of their effects on natural populations. We analysed how the recent sea surface temperature (SST) anomaly in the northeastern Pacific Ocean affects body condition, nutritional status, and immune competence of California sea lion pups. We found that pup body condition and blood glucose levels of the pups were lower during high SST events, although other biomarkers of malnutrition remained unchanged, suggesting that pups were experiencing early stages of starvation. Glucose-dependent immune responses were affected by the SST anomaly; specifically, pups born during high SST events had lower serum concentrations of IgG and IgA, and were unable to respond to an immune challenge. This means that not only were pups that were born during the SST anomaly less able to synthesize protective antibodies; they were also limited in their ability to respond rapidly to nonspecific immune challenges. Our study provides empirical evidence that atypical climatic conditions can limit energetic reserves and compromise physiological responses that are essential for the survival of a marine top predator.

## Introduction

During the boreal winter of 2013 a sudden increase in sea surface temperature (SST) was detected in the Gulf of Alaska [1,2]. This anomaly, referred to as ‘The Blob’, extended gradually southwards. The SST anomaly was further impacted by an El Niño event, confirmed in June 2015 [3]. These events led to an unprecedented 2 to 5°C increase above the historical average in the SST [2]. By summer 2015 the anomalously high SST had encompassed the entire northeastern Pacific, including the southernmost tip of the Baja California Peninsula, Mexico. Specifically, the SST anomaly was on average 1°C higher in 2015 than in 2014 for the central part of Baja California, where the San Benito Archipelago is located, and in some months exceeded 4°C [4]. The duration of the anomalies was also extended in 2015, with six of the months reaching over two standard deviations of the SST recorded between 1988 and 2014 [5].

High SSTs alter the primary productivity of the marine environment, which in turn affects all trophic levels [6,7], with disastrous consequences for sea birds and marine mammals [8,9]. One of the most dramatic effects of high SST was observed during the 1982–1983 El Niño, which led to a marked reduction of the Galapagos fur seal (*Arctocephalus galapagoensis*) population [10]. The high SST driven by ‘The Blob’ has already led to ecosystem alterations and biomass changes, reductions in the abundance of fishes [11], and increased stranding events of young California sea lions (*Zalophus californianus*; hereafter CSL) [12,13]. ‘The Blob’ was even related to the occurrence of a large harmful algal bloom that affected CSL as well as other marine mammals [14].

The CSL is an otariid pinniped that is distributed throughout the northeast Pacific. Its coastal feeding habits [15,16] make it particularly vulnerable to declines in prey availability [8]. As adult female CSL tend to procure prey less than 100 km from their breeding sites [17], well within the area affected by ‘The Blob’, diminished prey availability forces them to extend their foraging trips in order to feed [17,18], and results in consumption of prey of lower nutritional and energetic value, such as shortbelly rockfish, instead of the typically preferred sardines or anchovies in areas adjacent to California [12]. Such behavioural modifications will likely have consequences for pregnant or lactating CSLs (which are already undergoing an energetically-demanding process) [19], as well as for their offspring.

If suboptimal maternal nutrition means that fewer resources are available for developing pups, then costly physiological processes, such as immune responses, could be hampered. Maintaining and deploying the immune effectors needed for survival is greatly demanding of an individual’s resources [20]. For instance, in the house sparrow, *Passer domesticus*, cell-based immune responses to a mitogen requires the investment of energy equivalent to that needed to produce half an egg [21], and during bouts of fever, each 1°C rise in the core temperature leads to a 10–15% increase in metabolic rate [22]. Taking into account the atypical oceanographic conditions brought on by ‘The Blob’ and El Niño, and its impact on adult female CSL foraging behaviour [18,23], and diet [12], it is reasonable to assume that newborn pups did not experience ‘normal’ nursing during the SST anomaly, in terms of the amount and quality of milk received. There is some indication that this is indeed the case, as CSL pups born in 2015 in the Mexican north Pacific had lower mass than those born in 2014, when the SST anomaly was less pronounced in that region [5,18]. While there are published studies on the effects of maternal condition, foraging requirements and fasting times on milk quality [24–26], we are unaware of any study on variations in quality of otariid maternal milk caused by atypical oceanographic conditions. However, seasonal hunger has been shown to lead to a temporary decrease in production [27] and quality [28] of human milk, and maternal nutritional status can hinder neonatal development, growth, and survival [29]. If this were the same for the CSL, the abnormally high SST could impact pup fitness severely, particularly as offspring rely entirely on maternal milk during their first six months of age, and continue to nurse for most of their first year [30].

Here, we studied whether CSL pups born during the anomalous SST events had fewer resources available to elicit adequate immune responses than pups born during a year with normal SST conditions. To address this research question, we estimated pup condition, quantified blood biomarkers of malnutrition, and examined different measures of immune function in pups born during the 2014 and 2015 breeding seasons (abnormal SST events), and we compared them to those of pups born during a year of normal SST conditions. Based on resource allocation theory [31,32], we predicted that pups with a higher body condition and better nutritional status would have *i*) more marked *in vivo* responses to a mitogenic challenge, *ii*) higher immunoglobulin concentrations, and *iii*) fewer blood values outside of the normal clinical range.

## Materials and methods

### Study area and species

**Fig 1:**
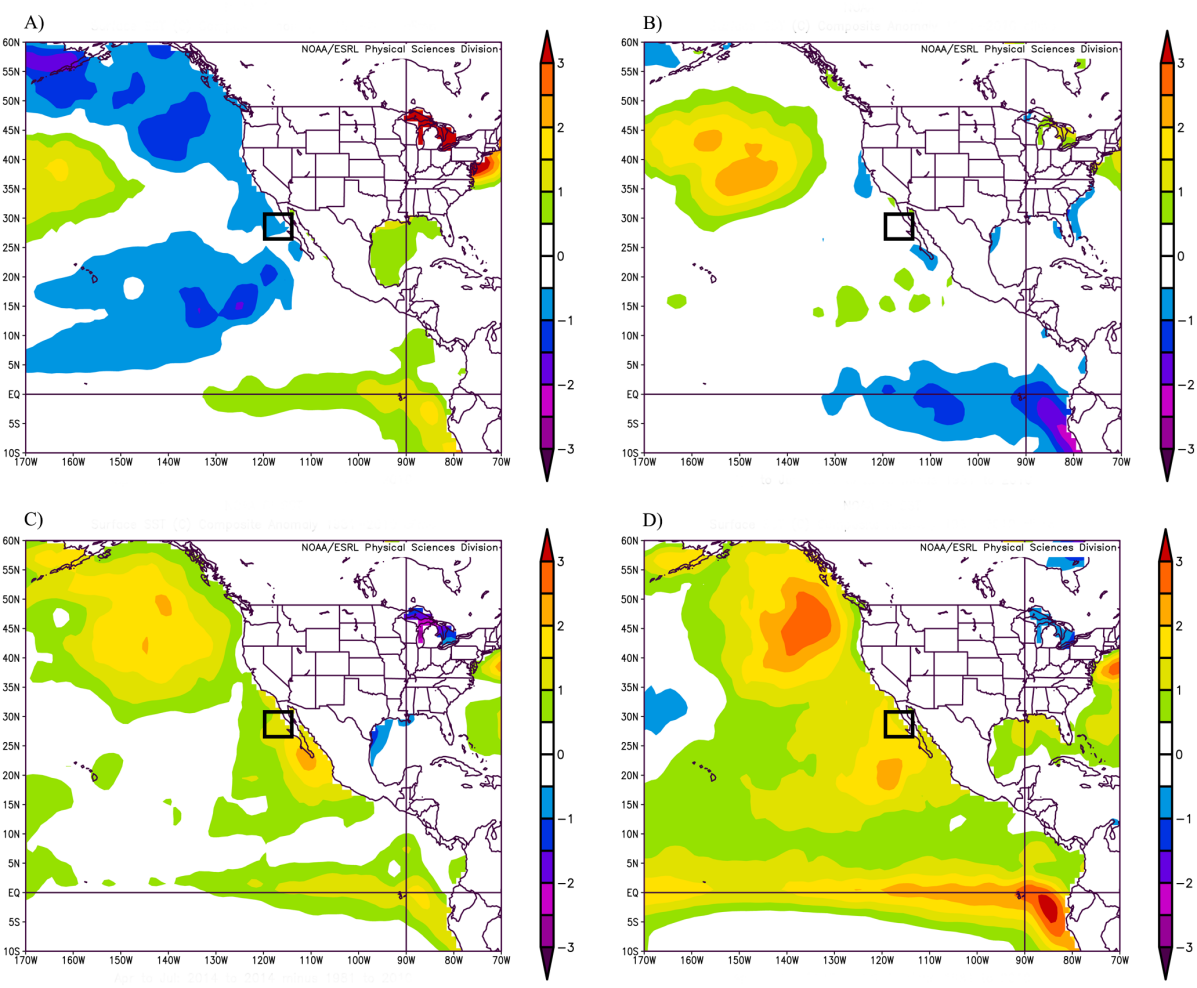
Sea surface temperature anomaly (°C) in the northeastern Pacific Ocean. Panels show the average of values recorded April 1^st^ to July 31^st^ for (**A**) 2012, (**B**) 2013, (**C**) 2014, and (**D**) 2015. The location of the San Benito Archipelago is indicated within the square. Images provided by the NOAA/ESRL Physical Sciences Division, Boulder Colorado from their Web site at http://www.esrl.noaa.gov/psd/. The plots were created by using data collected by the NCEP/NCAR Reanalysis Project [33].

As part of on-going studies on pinniped ecology, during the breeding season of 2014 (mid July) we sampled 28 CSL pups at the San Benito Archipelago (SBA; 28°18’N, 115°34’W) in the Mexican Northern Pacific. SBA is comprised by a group of three islands (named East, Middle, and West) located 270 km southeast of Guadalupe Island and 75 km northwest of the Baja California Peninsula, Mexico. All sampling was carried out in West Benito Island. In 2015, aware that the high SST associated with ‘The Blob’ and El Niño events had reached SBA, we returned to the field site and sampled 33 CSL pups born during that breeding season. However, even though the SST anomaly was less pronounced in 2014 than in 2015 as reported for the study region by the NCEP/NCAR Reanalysis Project [33] of the Earth System Research Laboratory, National Oceanic and Atmospheric Administration (NOAA), it certainly could not be considered a typical year (Fig 1). Thus, we considered 2014 as a ‘less-severe’ SST anomaly for the region. Since we did not have immune, haematological, and serological data from pups born at SBA during normal SST conditions, we used data from 23 clinically healthy CSL pups of the same age (6–8 weeks of age) born in 2012 at the rookery found in Granito Island (29°33’ N, 113°32’ W) as reference values. These pups were born during a year that was characterized by normal SST [5], and their capture, handling, and sampling as well as all haematological and serological analyses were conducted exactly as described above.

### Capture, handling and sampling

Sea lion pups were captured with hoop nets and were restrained manually during sampling. For each pup, mass and total length were recorded, and blood samples were collected. The mass was determined using a vertical hanging scale (0.1 kg precision) and the length from the tip of the tail to the nose was measured with a tape measure (1 mm precision).

Visual inspection by a veterinarian ascertained the general health status of each pup. Two blood samples (7 ml each) were collected from the caudal gluteal vein using an 18-gauge needle and vacuum tubes (Vacutainer, BD Biosciences, USA), one with EDTA and one without any preservative. Blood was kept in an upright position in a cooler, protected from direct sunlight, and was centrifuged at 3200 rpm for 10 min within three hours since the time of collection. Serum was separated and cryopreserved in a liquid nitrogen container until further analysis.

Sampling was conducted by approval of the Bioethics Committee and IACUC of the Autonomous University of Queretaro. All procedures were carried out under permits SGPA/DGVS/11744/13 and SGPA/ DGVS/00195/15 issued by the Dirección General de Vida Silvestre (DGVS) of the Secretaría de Medio Ambiente y Recursos Naturales (SEMARNAT).

### Body condition and nutritional status

We calculated mass per unit length and obtained the scaled mass index for each pup [34]. This proxy of body condition was used because the relationship between energetic reserves and body size is complex, and this measure takes into account the scaling between body components and body size [34].

We quantified the concentration of different blood biomarkers of malnutrition [35]. Namely, we measured glucose, triglycerides, total cholesterol, HDL, total proteins, albumin, creatinine, and blood urea nitrogen. Parameters were measured by spectrophotometry using an automated biochemistry analyser (Spin Lab 120, Spinreact) and commercially available kits (Spinreact S.A.U. Ctra Santa Coloma 7, E-17176 Sant Esteve de Bas, Spain). The biochemistry analyser was designed for human use and was calibrated prior to running the samples using a calibrator from the manufacturer. Two controls of lyophilized human serum were run together with the CSL samples for each assay. If a control failed, the assay was repeated.

### Assessment of immune parameters

#### White blood cell counts

Total white blood cell (WBC) counts were performed less than 8 h after blood collection using a haemocytometer (Optic Labor, CA, USA). We prepared three blood smears per sample and fixed them in 90% methanol before staining with Wright solution. For each smear, we determined the different WBC populations by counting the number of neutrophils, band neutrophils, hypersegmented neutrophils, lymphocytes, monocytes, eosinophils, and basophils in 100 cells. Absolute numbers for each leucocyte type were calculated by multiplying the total white blood cell count by the percentage of the each leucocyte type.

Based on the WBC reference values (mean ± 2 SD; S1 Table) previously calculated for clinically healthy pups born during normal SST conditions, we determined the presence (or absence) of clinical indications of disease, namely leucocytosis, neutrophilia, left-shift, monocytosis, lymphocytosis, lymphopenia, eosinophilia and basophilia [36].

#### Immunoglobulin concentrations

Immunoglobulin (Ig) isotypes A and M were quantified by indirect ELISA using mouse anti-dog IgA horseradish peroxidase (HPR) conjugate and mouse anti-dog IgM HPR as primary antibodies, and goat anti-mouse IgG-HPR, Novex) as a secondary antibody. The Mucosal Immunology Laboratory of the Veterinary School at Bristol University, UK provided the primary antibodies. For details on the protocols used see Supporting Information. IgG concentrations were measured with a protein A ELISA as reported previously [37], with slight modifications (see Supporting Information). Absorbance was measured in an ELISA microplate reader (BioRad, USA) at 450 nm. For each isotype, absorbance readings were interpolated on a standard curve using dog serum (Bethyl Laboratories, USA) as a reference. All reactions were run in triplicate.

#### PHA challenge

We examined the pups’ ability to react to intradermal injections of phytohemagglutinin (PHA), a plant-derived lectin that stimulates T lymphocyte mitogenesis [38] and causes local inflammation as a direct result of tissue damage [39]. The challenges were conducted as done previously [40]. Briefly, for each pup we measured the thickness of the webbing between the second and third digits of both hind flippers and inoculated 100 μl of 1 mg/ml of PHA intradermally into the right flipper, and a control (100 μl of sterile saline solution) into the left flipper. Webbing thickness at the inoculation site was measured after 4 h [40]. All measurements were taken in triplicate to the nearest 0.01 mm with a thickness gauge (Mitutoyo, USA). The response to PHA was calculated as the difference between the change in median thickness of the left flipper and the change in median thickness of the right flipper. This challenge was conducted only on pups born in 2015, and data was compared to that recorded for pups of the same age, born in 2012, which had been challenged using the same protocol.

### Statistical analyses

We initially explored our dataset graphically to establish the spread and distribution of the data. Continuous response variables that deviated from the normal distribution were examined with Cullen and Frey graphs to determine their distribution. We built a series of independent generalized linear models (GLM) to examine *i*) variations in the measured parameters amongst birth cohorts (2014: less-severe SST anomaly; 2015 severe SST anomaly; 2012 normal SST conditions), and ii) explore the relationship between immune-related variables and condition or biomarkers of nutritional/metabolic status. Response variables that had a beta distribution (IgG, IgA, IgM, triglycerides, and all white blood cell populations) were modelled with a quasibinomial error distribution and logit link. Dichotomous response variables (presence of leucocytosis, neutrophilia, left-shift, monocytosis, lymphocytosis, lymphopenia, eosinophilia and basophilia) were modelled with a binomial error distribution and logit link. All other variables were normally distributed and were modelled with a Gaussian error distribution. Tukey HSD tests were used to further examine differences in variables between years. Due to unequal sample size and asymmetric variance, differences in webbing thickness (measure of the response to the PHA challenge) of pups born in 2015 and 2012 were examined with a Kruskal-Wallis rank sum test. Contingency tables were built in order to investigate whether the prevalence of each clinical indicator of deviation from health varied amongst birth cohorts. We used Fisher exact tests to determine the level of significance. All analyses were performed in R version 3.3.1 [41].

## Results

### Variation in body condition and nutritional parameters

**Fig 2:**
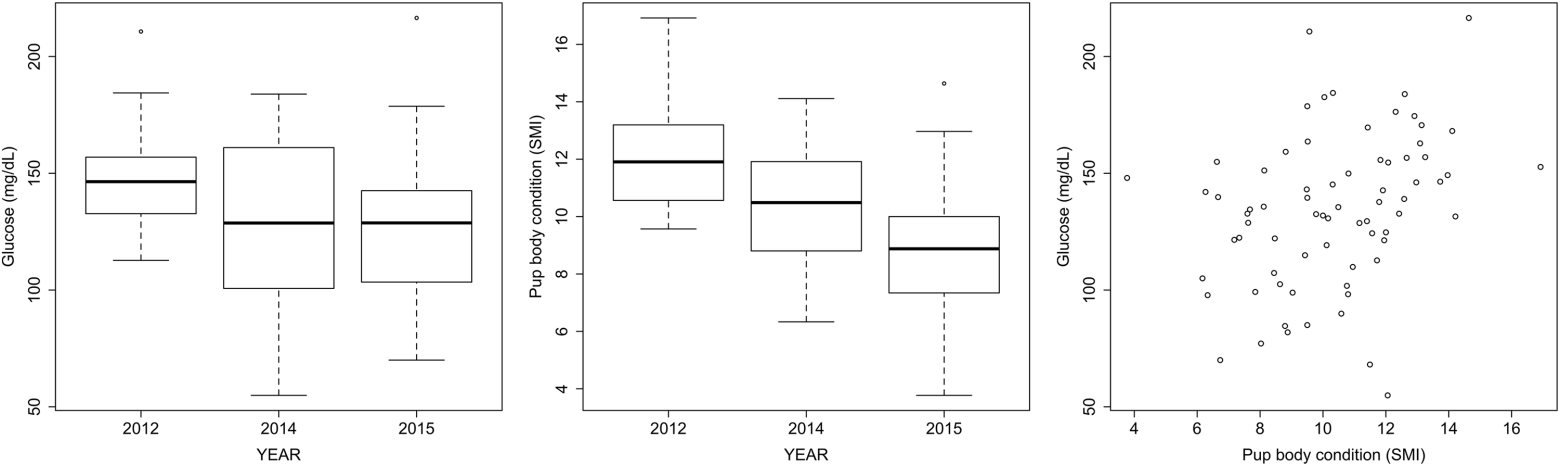
Indicators of nutritional status of California sea lion, *Zalophus californianus*, pups born during normal (2012) and atypical sea surface temperature conditions. A) Blood glucose concentration, B) Pup body condition calculated as the Scaled Mass Index, C) Blood glucose levels were related to pup body condition.

Blood levels of cholesterol, triglycerides, HDL, total protein, albumin, creatinine, and urea were within the reference values (S2 Table), and did not vary amongst birth cohorts. In contrast, mean glucose levels were 15% lower in pups born under atypical SST conditions (F_2.70_ = 4.35, p = 0.013; Fig 2a). The severity of the SST event did not affect the blood concentration of glucose (2014 = 2015; Tukey HSD, p = 0.14).

Body condition was significantly lower for pups born during the years of high SST conditions than during 2012 (F_2.79_ = 20.37, p = 7.35x10^-08^; Fig 2b), and those born in 2015 had a lower body condition than those born in 2014 (Tukey HSD, p = 0.02). Glycaemia was predicted by pup body condition, regardless of differences between years (F_2.71_ = 7.68, p = 0.007; Fig 2c). None of the other biomarkers of nutritional and metabolic status were related to body condition.

### Variation in immune parameters

**Fig 3:**
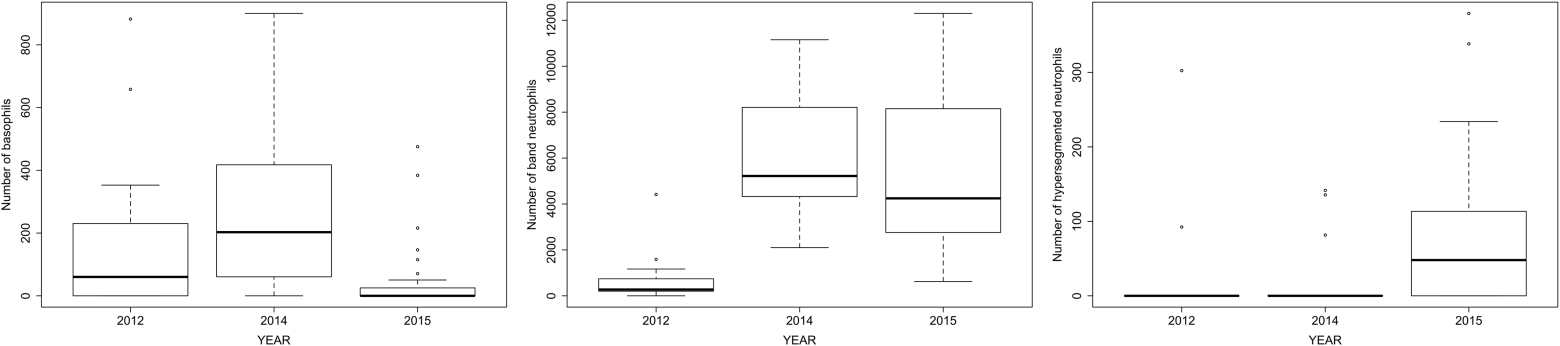
White blood cell counts of California sea lion, *Zalophus californianus*, pups that differed between those born during normal (2012) and atypical (2014 and 2015) sea surface temperature conditions. A) Basophils, B) Band neutrophils, C) Hypersegmented neutrophils.

Total WBC counts of the pups born at SBA were similar amongst birth cohorts, regardless of the severity of the SST anomaly, and did not differ from the values recorded for healthy pups of the same age that were born under normal environmental conditions (GLM; F_2,79_ = 0.26, p = 0.772). Most of the differential WBC counts of pups born in 2014 and 2015 were within the normal ranges recorded for healthy pups and did not vary markedly from those counts recorded for pups born in 2012 (S1 Table). However, basophils were virtually absent in pups born in 2015, compared to pups born in 2014 and 2012 (GLM; χ^2^_2,79_ = 4.84, p = 0.01; Fig 3a), and pups born in 2014 and 2015 had almost nine times more band neutrophils (mean = 5618.1) than pups born in 2012 (mean = 662.99) (GLM; χ^2^_2,79_ = 15.05, p = 2.2x10^-16^; Fig 3b). Furthermore, 50% of the pups born in 2015 had hypersegmented neutrophils, in contrast to what was observed for the other birth cohorts (2014: 0.8%; 2012: 0.92%), and these cells were significantly more abundant in pups born in 2015 (GLM; F_2,80_ = 28.52, p = 0.004; Fig 3c). None of the pups born in 2014 and 2015 had evidence of lymphopenia. The proportion of CSL pups with a left-shift was significantly higher for pups born during atypical years (2014: 100%, 2015: 90.6%, 2012: 4.3%; Pearson’s Chi-sq = 68.05; p = 0), and a similar pattern was seen for eosinophilia (2014: 29.6%, 2015: 21.9%, 2012: 4.3%; Pearson’s Chi-sq = 4.68; p = 0.02). The prevalence of leucocytosis, lymphocytosis, lymphopenia, monocytosis, neutrophilia, and basophilia did not vary amongst years.

**Fig 4:**
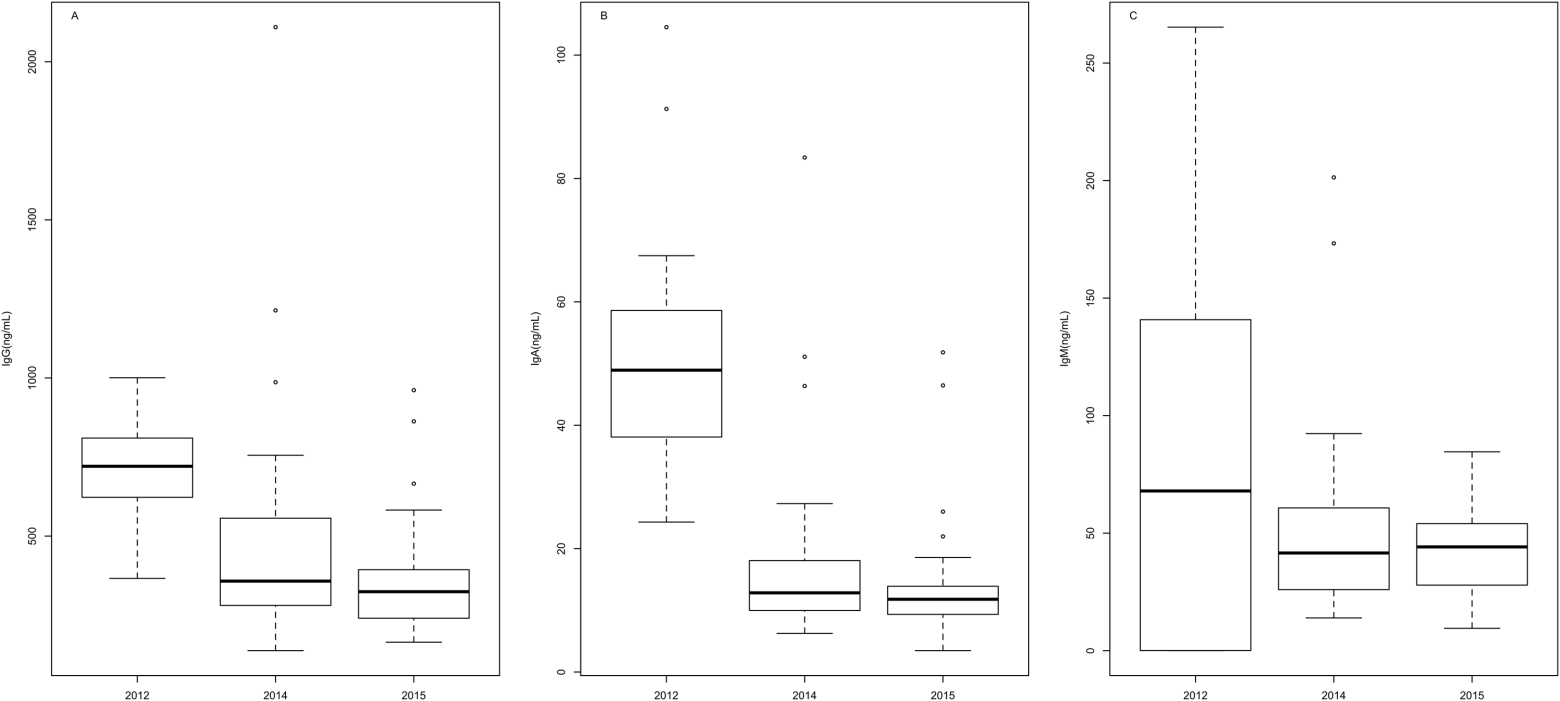
Serum antibody levels of California sea lion, *Zalophus californianus*, pups born under normal (2012) and atypical (2014 and 2015) sea surface temperature conditions. A) IgG, B) IgA, C) IgM.

IgM levels did not vary amongst birth cohorts, but the concentrations of IgG and IgA were significantly lower in pups born during an SST anomaly than in a normal year (GLM; IgG: χ^2^_2,70_ = 6.89, p = 0.002; IgA: χ^2^_2,70_ = 30.06, p = 3.77x10^-10^; Fig 4).

**Fig 5:**
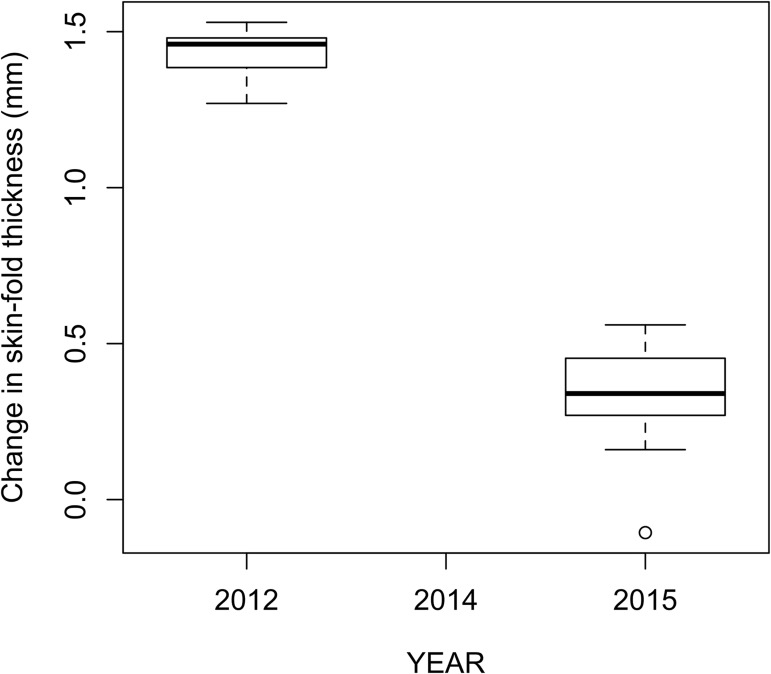
Skin-fold thickness in response to PHA challenge. Response to PHA in California sea lion, *Zalophus californianus*, pups born during normal (2012) and atypical (2015) sea surface temperature conditions.

The response to the PHA was 4.13 times lower for pups born in 2015 than in 2012 (Kruskal-Wallis; χ^2^ = 16.57, df = 1, p = 4.7x10^-5^; Fig 5).

### Body condition, nutritional status and immune effectors

Pups with a better body condition tended to have a higher serum concentration of IgA (GLM; F_1,70_ = 7.42, p = 0.008) and both IgG and IgA concentrations increased with blood glucose level (GLM; IgG: F_2,71_ = 12.86, p = 0.0006; IgA: F_2,71_ = 14.57, p = 0.0003). The response to PHA also increased in relation to blood glucose (GLM; F_2,72_ = 13.96, p = 7.55x10^-06^). None of the other biomarkers of nutrition were significantly associated with any of the immune effectors.

## Discussion

The immune system is indispensable for the survival of any individual. In order to function adequately, the effectors that participate in mounting an immune response require nutrients and energy [20,32]. Here, we tested the hypothesis that if the abnormally high SST conditions caused by ‘The Blob’ [1,2], and a simultaneous El Niño event [3,5] posed a nutritional and energetic challenge for pregnant and lactating CSL females [17,18], then their offspring were likely to have lower body condition, altered nutritional status and impaired immune responses. As we did not have data for pups born at SBA during normal SST conditions, we used data collected from pups of the same age that were born during a normal year at a rookery located in the midriff region of the Gulf of California. While not ideal, this data was considered valid to use as reference data, as published data on haematology, blood chemistry, and serology of CSL focuses mainly on older animal, and mostly on stranded or captive individuals [42].

We found that body condition was significantly lower in pups born during the atypical climatic conditions, and was even lower for pups born in 2015, when the SST was higher than in 2014 [3,5]. However, except for glucose, which was 15% lower in pups born under atypical SST conditions, the biomarkers selected to assess nutritional/metabolic status were within the reference values used in this study and also fell within the range reported for young CSL [36]. This implies that rather than malnutrition, these pups may have experienced undernutrition, an early stage of starvation in which an individual has not yet switched to gluconeogenesis, that if not corrected will lead to malnutrition [43]. Finding that glycaemia was directly predicted by pup body condition further strengthens this possibility.

None of the pups showed any clinical signs of disease at physical examination, and most differential WBC counts were within the reference values here used (pups born in Granito in 2012), as well as within the normal range reported for yearling CSL [36], suggesting that at the time of sampling, the health of the pups was comparable amongst birth cohorts. One exception was eosinophils, which tended to be more abundant in pups born in 2014 and 2015 than those born in 2012. Eosinophilia has been related to domoic acid intoxication [44], and it could be argued that the CSL pups born during the high SST conditions were affected by mammary transmission of domoic acid from lactating mothers that fed in areas where toxic blooms of domoic acid-producing algae, *Pseudo-nitzschia* spp., were occurring [45]. The high SST conditions of 2014 and 2015 are known to have favoured blooms of *Pseudo-nitzschia australis* along the western coast of the US, causing considerable damage to CSL and other marine mammals [14]. However, to the best of our knowledge, there has been no evidence of domoic acid intoxication in sea lions at SBA, nor have blooms of *Pseudo-nitzschia* been reported to occur within the feeding range of SBA adult females during the 2014 and 2015 anomalies. Also, during the duration of our field trip we failed to observe abortions, as would be expected during *in utero* toxicity if pregnant females fed in locations where *Pseudo-nitzschia* blooms were occurring [46]. Except for one adult male that was displaying an unusual “rolling” movement along the beach, we did not observe seizures or altered behaviours associated with DA intoxication [47] in any adult female, juvenile or pup during the study years. In that sense, while possible, it is unlikely that the CSL pups were affected by DA intoxication.

In addition to eosinophilia, the second exception to the largely normal leukogram was the number of basophils, which were virtually absent in pups born in 2015, compared to pups born in 2014 and 2012. While circulating basophil numbers are typically low compared to other WBC types, an absence of basophils can be indicative of adrenocortical hyperfunction due to stress [48]. It is possible that pups born in 2015 might have experienced high levels of stress due to suboptimal nursing, or could have been affected *in utero* by maternal levels of stress [49], partially explaining the unusually high number of abortions recorded at SBA at the beginning of May 2015, prior to the breeding season (unpublished data). Furthermore, pups born at SBA in 2014 and 2015 had almost nine times more band neutrophils than pups born in 2012. The presence of these immature cells tends to indicate that the demand for neutrophils exceeds its supply. As we found no clinical evidence of malaise, and the other WBC populations in these pups were within the ranges, it is possible that their presence was an early indication of subclinical infection [50]. Alternatively, the high numbers of band neutrophils could be undernutrition, as has been reported for starving juvenile Northern elephant seals, *Mirounga angustirostris* [51]. The fact that half of the pups born in 2015, during the most severe high SST event, had hypersegmented neutrophils which are common during nutrient-deficiency anaemia in human neonates [52], would appear to support this possibility.

We hypothesized that if pups were experiencing suboptimal nutrition, immunoglobulin synthesis might be constrained. We found evidence to support this, as the concentration of IgG and IgA (but not IgM) was markedly lower in pups born during ‘atypical’ years. Given that pup blood glucose levels were found to be altered by the high SST events, the observed pattern was exactly as would be predicted because class-switching of IgG and IgA occurs via T-cell dependent (glucose-dependent) and T-cell independent pathways [53], while IgM class switching is T-cell independent [54].

If nutritional and energetic impoverishment impairs pups’ body condition and limits glucose-dependent immunoglobulin class switching, other T-cell dependent immune responses could also be affected. Indeed, we found that responses to a mitogenic challenge were severely impaired in individuals born under atypical environmental conditions. The apparent inability of these pups to respond to PHA is most likely due to insufficient energetic reserves, as was evidenced by the direct association between the magnitude of the swelling response to PHA and blood glucose concentration. This means that not only were the pups that were born during the SST anomaly less able to synthesize protective antibodies; they were also limited in their ability to respond rapidly to nonspecific immune challenges. Taken together, these results constitute evidence that atypical climatic conditions can encumber the energetic reserves and immune competence of neonates, thus decreasing their fitness.

## Conclusions

The consequences of having suboptimal immune responses could be devastating to the entire CSL 2014 and 2015 cohorts, particularly if a highly virulent pathogen were to ‘enter’ the population. Even in absence of such a challenge, without proper nutrition, pups are likely to be unable to elicit responses against opportunistic pathogens, and mortality would expectedly be high. We returned to SBA in February 2016 and found that the entire CSL colony was reduced by 77%, relative to the previous year during the same dates (total count: 584 in 2016 *vs*. 2,555 in 2015). Extremely few live pups were found at the archipelago (343 in 2016 *vs*. 1,567 in 2015) and most of those that were present were severely emaciated and weak. An unusual high number of carcasses were also recorded. It is too early to understand the impacts that the abnormal oceanographic conditions will have on CSL pups later in their life, but based on life-history theory, their fitness would expectedly be poor. Furthermore, as the high SST conditions encompassed the northeastern Pacific, the entire CSL population could be impacted, as has been reported for other pinnipeds during past El Niño events [10].

In conclusion, we have shown that abrupt and unexpected environmental changes can affect key physiological components. The observed effects of the abnormally high SST linked to The Blob and El Niño are unlikely to be limited to the CSL; the entire northeastern Pacific ecosystem could potentially be vulnerable. Under the current climatic scenario, systematic surveys within this region would be invaluable to determine the full scope of effects and assess their wider and long-term consequences.

## Supporting information

S1 Table Reference values of total and differential white blood cell (WBC) counts from clinically healthy California sea lion, *Zalophus californianus*, pups born in 2012 at Granito Island in the Gulf of California. The table also shows the mean and standard deviation of each cell type for pups born in 2014 and 2015 at the San Benito Archipelago.
(PDF)

S2 Table Reference values of blood chemistry parameters from clinically healthy California sea lion, *Zalophus californianus*, pups born in 2012 at Granito Island in the Gulf of California. The table also shows the mean and standard deviation of each cell type for pups born in 2014 and 2015 at San Benito Archipelago.
(PDF)

S1 File Data used for the analyses presented in this paper. (PDF)

S2 File More detailed description of methods used. (PDF)
