# Supplementary material for: Climatic anomaly affects the immune competence of California sea lions
Source: PLoS One. 2017 Jun 28;12(6):e0179359. doi: 10.1371/journal.pone.0179359 (PMC5489150; doi:10.1371/journal.pone.0179359)
Supplement: S1 Table — The table also shows the mean and standard deviation of each cell type for pups born in 2014 and 2015 at the San Benito Archipelago. (PDF) [file pone.0179359.s001.pdf]

S1 Table. Reference values of total and differential white blood cell (WBC) counts from clinically healthy California sea lion, *Zalophus californianus*, pups born in 2012 at Granito Island in the Gulf of California. The table also shows the mean and standard deviation of each cell type for pups born in 2014 and 2015 at the San Benito Archipelago.

	**2012**	**2014**	**2015**
**WBC**	4500-19529	11716 ± 4008	11145 ± 5308
**Total neutrophils**	1359-9284	7066 ± 3352	6779 ± 3597
**Segmented neutrophils**	1025-13824	1240 ± 1130	1083 ± 647
**Band neutrophils**	0-1403	5813 ± 2488	5618 ± 3147
**Hypersegmented neutrophils**	0-144	13 ± 39	78 ± 111
**Lymphocytes**	273-4271	2831 ± 1176	2747 ± 1924
**Monocytes**	0-1772	744 ± 382	710 ± 452
**Eosinophils**	0-1101	821 ± 650	839 ± 759
**Basophils**	0-660	253 ± 247	71 ± 171
