# Supplementary material for: Climatic anomaly affects the immune competence of California sea lions
Source: PLoS One. 2017 Jun 28;12(6):e0179359. doi: 10.1371/journal.pone.0179359 (PMC5489150; doi:10.1371/journal.pone.0179359)
Supplement: S2 Table — The table also shows the mean and standard deviation of each cell type for pups born in 2014 and 2015 at San Benito Archipelago. (PDF) [file pone.0179359.s002.pdf]

S2 Table. Reference values of blood chemistry parameters from clinically healthy California sea lion, *Zalophus californianus*, pups born in 2012 at Granito Island in the Gulf of California. The table also shows the mean and standard deviation of each cell type for pups born in 2014 and 2015 at San Benito Archipelago.

	2012	2014	2015
**Glucose (mg/dL)**	105-196	128 ± 35	126 ± 31
**Cholesterol (mg/dL)**	126-200	158 ± 38	173 ± 46
**Triglycerides (mg/dL)**	0-116	64 ± 37	67 ± 30
**High density lipoprotein (mg/dL)**	77-155	103 ± 21	117 ± 23
**Total protein (g/dL)**	5.1-6.7	6.2 ± 0.6	6.1 ± 0.6
**Albumin (g/dL)**	3.3-4.0	3.7 ± 0.2	3.7 ± 0.3
**Creatinine (mg/dL)**	0.2-0.6	0.5 ± 0.2	0.4 ± 0.1
**Blood urea nitrogen (mg/dL)**	16-52	42 ± 16	39 ± 17
