# Supplementary material for: Climatic anomaly affects the immune competence of California sea lions
Source: PLoS One. 2017 Jun 28;12(6):e0179359. doi: 10.1371/journal.pone.0179359 (PMC5489150; doi:10.1371/journal.pone.0179359)
Supplement: S1 File — (PDF) [file pone.0179359.s003.pdf]

Supporting information – S1 File

Supplementary methods

Measurement of immunoglobulin concentrations

Immunoglobulin (Ig) isotypes A, M and G were quantified by indirect ELISA (for detailed protocol used see Supplementary Material). For IgA and IgM, 96-well microplates (Nunc-Immunoplate–Maxisorp USA) were coated with 50 µl of CSL serum that contained 10 µg/ml of protein in carbonate buffer pH 9.6, and incubated for 2 h at 37°C. After two washings with 100 µl phosphate buffered solution containing 0.05% Tween 80 (PBST), the plates were treated with 100 µl of blocking solution (5% non-fat powdered milk in PBST) for 1 h at 37 °C. Following four washings with PBST, plates were incubated with 100 µl of primary antibody in 5% milk solution overnight at 4°C (IgA assays used 1:2,000 dilution of mouse anti-dog IgA horseradish peroxidase (HPR) conjugate; IgM assays used 1:4,000 dilution of mouse anti-dog IgM horseradish peroxidase conjugate. Antibodies were obtained from the Mucosal Immunology Laboratory of the Veterinary School at Bristol University, UK. Plates were washed twice and the secondary antibody (1:16,000 dilution of goat anti-mouse IgG-HPR, Novex) was added, and incubated at room temperature for 1 h. Plates were washed five times with PBS-T to remove unbound secondary antibody and we added 100 µl/well of substrate [3, 3, 5, 5-Tetramethyl-benzidine, (TMBE, Sigma) 10 mg; 0.003% H2O2]) in citrate buffer, 0.5 M, pH 5.0 and allowed 15 min incubation. We stopped the reaction by adding 100 µl/well of 1N HCL.

We measured immunoglobulin G (IgG) concentrations with a protein A ELISA as reported previously [1], but with slight modifications: namely, we coated the microplates as we did for IgA and IgM assays, substrate TMBE was allowed to react for 5 min and the reaction was stopped by adding 100 µl/well of 1N HCL. Absorbance was measured in an ELISA microplate reader (BioRad, USA) at 450 nm. For each isotype, absorbance readings were interpolated on a standard curve using dog serum (Bethyl Laboratories, USA) as a reference. All reactions were run in triplicate.

1. Hall AJ, McConnell BJ, Barker RJ. The effect of total immunoglobulin levels, mass and condition on the first-year survival of Grey Seal pups. Funct Ecol. 2002;**16**: 462-474. doi:10.1046/j.1365-2435.2002.00649.x
