# Supplementary material for: Climatic anomaly affects the immune competence of California sea lions
Source: PLoS One. 2017 Jun 28;12(6):e0179359. doi: 10.1371/journal.pone.0179359 (PMC5489150; doi:10.1371/journal.pone.0179359)
Supplement: S2 File — (PDF) [file pone.0179359.s004.pdf]

Supporting information – S2 File
Raw data

ID	YEAR	SMI	GLUCOSE	TRIGLYCERIDES	HDL	CHOLESTEROL	ALBUMIN	CREATININE	TOTAL.PROTEIN	UREA
14BE01	2014	10.58	89.9	74.5	132.1	217	3.9	0.4	6.7	41.8
14BE02	2014	8.63	102.5	57	133.2	220.7	3.8	0.5	6.1	42.9
14BE04	2014	10.95	109.9	53.8	132.1	166.5	3.9	0.4	6.1	40.7
14BE05	2014	7.68	134.5	48.6	93.1	139.9	3.5	0.9	7.8	52.4
14BE06	2014	11.42	169.6	159.4	119.9	176.2	3.4	1	6.7	47.6
14BE07	2014	12.00	124.7	61.7	91.4	122.8	3.4	0.1	5	36.4
14BE08	2014	9.52	163.6	46	64.9	101	3.6	0.4	5.9	29.2
14BE09	2014	12.06	54.9	54.6	98.3	144.8	3.9	0.4	6.1	35.1
14BE10	2014	7.60	132.7	62	96.6	227.9	4	0.5	7	52.7
14BE11	2014	8.44	107.3	34.9	94.6	180.2	3.7	0.6	6.3	50.4
14BE12	2014	8.81	159.2	50	104.3	152.4	3.6	0.5	5.8	31.2
14BE13	2014	9.03	98.9	48.4	129.4	199	3.8	0.4	6.2	22.7
14BE14	2014	10.49	135.5	55.7	89.9	159.1	3.8	0.4	7	29.1
14BE15	2014	8.47	122.1	35	85.6	111.1	3.4	0.4	5.3	43
14BE16	2014	8.79	84.6	40.9	139.4	224.1	3.8	0.4	6.2	38.5
14BE17	2014	9.40	NA	NA	NA	NA	NA	NA	NA	NA
14BE18	2014	10.13	NA	NA	NA	NA	NA	NA	NA	NA
14BE19	2014	11.08	NA	NA	NA	NA	NA	NA	NA	NA
14BE20	2014	12.31	176.3	142.6	88.1	118.9	3.7	0.4	5.7	49.4
14BE21	2014	13.09	162.8	36.9	99.5	140.6	3.7	0.6	6	32.4
14BE22	2014	9.65	NA	NA	NA	NA	NA	NA	NA	NA
14BE23	2014	12.60	183.9	62.5	93.1	120.4	3.6	0.4	7.1	45.2
14BE24	2014	11.83	155.7	40.1	86	121.5	3.5	0.4	5.8	16.4
14BE25	2014	NA	122.1	55	71.8	186.9	3.9	1.1	5.9	51.1
14BE26	2014	12.08	154.6	78.2	101.7	146.1	3.7	0.3	6.6	104.3
14BE27	2014	11.49	68.1	171	133.4	170.9	3.7	0.2	6	52.1
14BE28	2014	14.11	168.1	37.7	103.8	136.3	3.8	0.5	5.9	27.5
14BE30	2014	6.33	97.8	40.2	78.9	111.8	3.5	0.5	6.1	35
15BE01	2015	10.16	130.7	46.7	102.8	134.7	3.6	0.5	6	50
15BE02	2015	6.26	142	59	84.5	104.4	3.4	0.4	5.4	23
15BE03	2015	6.63	154.9	46.3	113.8	212	3.8	0.4	5.8	29.3
15BE04	2015	9.04	NA	NA	NA	NA	NA	NA	NA	NA
15BE05	2015	11.16	128.7	99.1	96.6	127.3	3.7	0.2	5.6	94.4
15BE06	2015	11.95	121.3	48.8	78.8	119.8	3.6	0.4	5.8	39.3
15BE07	2015	8.88	81.9	53.6	135.8	179.6	3.6	0.4	5.6	30.5
15BE08	2015	6.66	139.8	79	121.7	162	3.5	0.4	5.8	50.6
15BE09	2015	8.17	NA	NA	NA	NA	NA	NA	NA	NA

Supporting information – S2 File

Raw data

15BE10	2015	12.97	146.1	41.4	162.5	207.2	3.6	0.5	6	31.8
15BE11	2015	6.73	70	33.3	117.3	141.5	3.6	0.4	5.7	23.5
15BE13	2015	7.11	NA	NA	NA	NA	NA	NA	NA	NA
15BE14	2015	6.17	105	39.4	138	217.7	3.9	0.2	6.1	29.3
15BE15	2015	7.85	99.2	40.2	102.4	142.9	3.6	0.5	5.7	33
15BE16	2015	7.62	128.8	80.9	117.2	169.8	4.2	0.3	6.7	46
15BE17	2015	7.34	122.4	64	137.1	236.9	3.9	0.4	6.8	27.4
15BE18	2015	10.12	119.2	82.6	101.4	153.2	3.5	0.2	5.5	76.6
15BE19	2015	7.18	121.5	42.1	110	144.6	3.7	0.4	5.9	29.1
15BE20	2015	9.50	85	49.6	147.7	188.2	3.9	0.4	6.2	32.2
15BE21	2015	10.00	131.9	50.3	109.8	128.7	3.8	0.4	6.3	40.6
15BE22	2015	8.12	135.7	98.2	101	197	3.7	0.6	5.4	31.2
15BE23	2015	10.80	98.2	36.7	93.6	128.1	3.5	0.2	5.5	29.8
15BE24	2015	9.37	NA	NA	NA	NA	NA	NA	NA	NA
15BE25	2015	8.03	77.1	126.2	147.4	278.3	3.8	0.4	6.7	66.9
15BE26	2015	3.77	148	165.9	140.9	254.4	3.7	0.5	6.4	52.2
15BE27	2015	8.13	151.2	65	95.2	135.9	3.4	0.5	5.8	25.5
15BE28	2015	9.50	139.5	67.3	106.1	225.2	3.6	0.3	5.8	25
15BE29	2015	9.43	114.9	34.6	109.5	147.7	3.5	0.5	5.8	29.9
15BE30	2015	9.49	143.1	56.9	96.9	168	3.6	0.4	6.3	34.7
15BE31	2015	14.64	216.5	99.7	86.7	116.1	4.7	0.3	6.9	35.1
15BE32	2015	10.76	101.8	77.9	160.7	235.1	3.8	0.3	8	52.7
15BE33	2015	8.33	NA	NA	NA	NA	NA	NA	NA	NA
15BE34	2015	9.50	178.7	77.3	152.6	197.9	3.6	0.6	6.2	21.6
12GR02	2012	13.25	156.9	50.6	124.4	161.6	3.8	0.5	6.1	37.3
12GR03	2012	12.42	132.7	51.6	118.7	185.8	3.8	0.4	6	43.5
12GR04	2012	14.53	NA	NA	NA	NA	NA	NA	NA	NA
12GR05	2012	10.81	149.9	40.4	146.2	170.7	3.8	0.4	5.7	35.4
12GR06	2012	12.58	139	36.1	116.5	159.9	3.9	0.4	6.2	26.4
12GR07	2012	9.57	210.7	37.5	106.9	139.6	3.9	0.3	5.8	31.5
12GR08	2012	12.66	156.6	45.6	128.6	173.1	3.8	0.6	6.4	22.4
12GR09	2012	11.72	112.7	51.6	104.6	142.3	3.6	0.4	6.2	25.9
12GR10	2012	11.56	124.3	34.9	78.9	167.6	3.9	0.4	6.3	24.1
12GR11	2012	16.92	152.7	46.5	144.7	175.8	3.7	0.4	5.1	26.9
12GR12	2012	10.27	NA	NA	NA	NA	NA	NA	NA	NA
12GR14	2012	10.31	145.2	49.2	97.5	151.9	3.6	0.4	5.8	24.8
12GR15	2012	14.21	131.5	50.6	107	143.6	3.8	0.5	6.2	31.7
12GR17	2012	13.14	170.6	123.8	83.8	141.2	3.6	0.3	6.2	48.9

Supporting information – S2 File

Raw data

12GR18	2012	13.73	146.4	79.7	110.1	168.8	3.8	0.6	5.6	38.9
12GR20	2012	10.32	184.4	36.6	158.8	203.7	3.7	0.4	6.2	27.4
12GR22	2012	11.79	137.7	40.2	126.4	158.1	3.6	0.5	6.4	22.4
12GR23	2012	12.91	174.5	87.4	117.6	167.5	3.6	0.2	5.6	46.1
12GR24	2012	11.91	142.7	125.2	106.4	166.2	3.9	0.3	6	45.3
12GR25	2012	10.04	182.6	47.8	116.4	153.8	3.5	0.3	6.3	48.8
12GR27	2012	11.40	129.5	112.4	102.6	148.3	3.5	0.2	5.7	45.3
12GR28	2012	9.78	132.5	31.4	100	142.8	3.2	0.3	4.9	23.2
12GR29	2012	13.97	149.2	28.8	137.2	206.5	3.8	0.4	5.9	40.6

Supporting information – S2 File

Raw data

IGG	IGA	IGM	CMI	WBC	MONOCYTES	TAL.NEUTROPH	MENTED.NEUTRO	AND.NEUTROPHI	GMENTED.NEUT
519.43	16.49	46.43	NA	NA	NA	NA	NA	NA	NA
622.64	6.84	23.85	NA	9000	720	5580	360	5220	0
137.82	12.53	20.35	NA	12840	385	8860	642	8218	0
1213.71	46.36	201.35	NA	17520	1051	10687	701	9986	0
2109.43	83.40	173.27	NA	13560	949	7594	1220	6238	136
407.82	10.68	70.18	NA	6000	540	3840	240	3600	0
755.68	19.25	92.35	NA	12480	1498	4992	998	3994	0
230.14	6.71	43.18	NA	14160	1274	10054	991	8921	142
298.71	16.88	28.43	NA	8160	898	5304	408	4814	82
354.96	14.64	39.93	NA	12240	612	5018	367	4651	0
270.50	9.57	27.43	NA	9360	1685	2714	94	2621	0
586.39	21.13	67.02	NA	14760	886	5904	1181	4723	0
308.00	10.94	35.10	NA	7560	1058	2948	0	2948	0
463.54	15.09	49.02	NA	9000	360	4320	540	3780	0
280.14	6.26	25.52	NA	6360	700	2608	509	2099	0
NA	NA	NA	NA	6840	205	4651	616	4036	0
NA	NA	NA	NA	8160	571	6365	1142	5222	0
NA	NA	NA	NA	4560	274	2417	502	1915	0
281.39	10.38	26.43	NA	11280	1241	6091	1128	4963	0
360.32	14.12	54.43	NA	15480	619	9752	1548	8204	0
NA	NA	NA	NA	9480	379	6352	1043	5309	0
986.57	27.30	75.10	NA	20280	1217	14602	3448	11154	0
364.25	12.17	21.02	NA	14520	436	10164	3485	6679	0
176.04	6.42	54.18	NA	12120	485	8848	1212	7636	0
335.68	11.68	20.77	NA	13080	523	7586	1439	6148	0
300.86	13.14	50.60	NA	17040	682	13291	4090	9202	0
180.68	8.89	13.93	NA	11760	470	6586	1646	4939	0
526.93	51.10	32.18	NA	18720	374	13666	3931	9734	0
390.14	13.31	53.43	NA	3480	487	1984	557	1392	35
665.86	10.25	13.68	NA	4320	130	2333	346	1858	130
293.18	18.56	16.60	NA	8040	1849	3136	563	2492	80
NA	NA	NA	NA	9840	1279	5412	1476	3936	0
362.64	12.33	40.18	NA	5400	540	2916	702	2106	108
276.39	9.51	68.02	NA	4800	624	1344	672	624	48
221.93	5.74	9.52	0.29	7800	702	4836	780	3822	234
862.82	46.45	84.60	-0.106	6960	766	4176	626	3480	70
NA	NA	NA	0.46	12600	756	9450	1638	7434	378

Supporting information – S2 File
Raw data

576.75	14.51	47.02	0.49	11880	713	8554	1426	7009	119
273.00	11.65	17.77	0.44	5400	324	3402	864	2484	54
NA	NA	NA	0.53	7200	504	5112	1296	3816	0
297.46	13.27	26.02	0.466	7080	283	3540	283	3186	71
213.00	7.88	48.07	0.3	6360	191	4198	572	3625	0
280.32	9.25	49.14	0.56	5040	202	2621	554	1966	101
216.21	9.44	54.68	0.223	11520	1382	8525	1498	7027	0
401.93	10.45	66.46	0.16	10800	756	3780	756	3024	0
357.11	12.92	34.50	0.4	15360	1075	10906	1997	8909	0
211.93	3.47	44.14	0.42	14640	586	6442	1903	4538	0
397.46	9.60	84.23	0.36	22200	1776	11322	2664	8658	0
197.29	12.40	16.91	0.29	16200	1458	11664	1782	9882	0
252.11	5.68	46.02	0.47	7320	805	4758	512	4246	0
NA	NA	NA	0.1	15960	160	8299	1596	6703	0
164.43	6.26	39.95	0.32	24600	492	15006	2706	12300	0
322.11	11.94	35.77	0.25	16680	167	11342	1501	9674	167
961.39	26.00	62.35	NA	11280	1015	7783	451	6994	338
228.54	12.95	24.10	NA	10920	437	8408	764	7644	0
369.25	10.84	39.52	NA	18960	569	12703	948	11376	379
367.46	21.97	29.77	NA	13320	533	7193	266	6926	0
582.29	17.98	44.93	NA	8280	331	4802	580	4223	0
326.21	8.95	NA	NA	17280	1210	11750	864	10714	173
NA	NA	NA	NA	15120	605	9223	1512	7711	0
358.89	51.81	64.93	NA	NA	NA	NA	NA	NA	NA
768.80	53.87	262.09	1.4	8800	264	6776	6512	264	0
484.76	33.97	180.27	NA	9600	0	7680	7680	0	0
897.34	104.53	0.02	NA	7420	0	3116	1781	1336	0
386.93	28.55	0.07	1.37	11520	346	8410	8179	230	0
880.32	60.49	265.27	NA	10220	204	7052	6030	1022	0
517.34	36.95	NA	1.46	13580	0	10321	10049	272	0
624.63	53.97	0.07	NA	13160	132	6712	6712	0	0
765.86	52.74	NA	NA	12040	602	8187	7826	361	0
828.18	50.82	NA	NA	13720	0	11388	11113	274	0
640.97	48.92	NA	NA	8400	420	3528	3276	252	0
620.62	45.99	78.19	NA	10360	1140	6112	5284	829	0
1000.77	91.27	NA	NA	7420	445	2968	2226	742	0
633.09	40.97	0.05	NA	10080	302	6048	5846	202	0
791.48	63.27	90.40	NA	9240	370	5636	5452	185	92

Supporting information – S2 File

Raw data

378.09	24.30	NA	NA	6020	181	3793	3732	60	0
662.69	48.39	0.02	1.27	14700	588	7938	7644	294	0
869.88	64.83	NA	NA	13860	1109	10118	9841	277	0
791.84	44.18	0.04	NA	12320	986	7762	7762	0	0
366.21	35.04	140.80	NA	16680	1835	11009	9841	1168	0
909.52	67.48	NA	NA	15120	605	9223	8921	302	302
786.84	56.73	75.40	1.41	22920	2521	17190	16273	917	0
720.77	39.23	60.51	1.53	11520	691	8064	7142	922	0
652.11	33.52	NA	1.48	17640	353	13230	11642	1588	0

Supporting information – S2 File
Raw data

EOSINOPHILS	BASOPHILS	LYMPHOCYTES	Leucocytosis	Leucopenia	Neutrophilia	Left.shift	Lymphocytosis	Monocytosis	Eosinophilia
NA	NA	NA	NA	NA	NA	NA	NA	NA	NA
90	450	2160	0	0	0	1	0	0	0
642	385	2568	0	0	0	1	0	0	0
526	350	4906	1	0	0	1	1	0	0
678	136	4204	0	0	0	1	0	0	0
660	0	960	0	0	0	1	0	0	0
1872	499	3619	0	0	0	1	0	0	1
991	0	1841	0	0	0	1	0	0	0
571	0	1387	0	0	0	1	0	0	0
1102	734	4774	0	0	0	1	1	0	1
1217	187	3557	0	0	0	1	0	0	1
2952	738	4280	0	0	0	1	1	0	1
1966	227	1361	0	0	0	1	0	0	1
990	900	2430	0	0	0	1	0	0	0
1145	0	1908	0	0	0	1	0	0	1
68	342	1573	0	0	0	1	0	0	0
408	0	816	0	0	0	1	0	0	0
228	182	1459	0	0	0	1	0	0	0
564	0	3384	0	0	0	1	0	0	0
774	464	3870	0	0	0	1	0	0	0
95	0	2654	0	0	0	1	0	0	0
1014	203	3245	1	0	1	1	0	0	0
1162	145	2614	0	0	0	1	0	0	1
121	121	2545	0	0	0	1	0	0	0
392	0	4578	0	0	0	1	1	0	0
170	341	2556	1	0	1	1	0	0	0
470	235	3998	0	0	0	1	0	0	0
1310	187	3182	1	0	1	1	0	0	1
244	0	766	0	1	0	0	0	0	0
562	475	821	0	0	0	1	0	0	0
161	0	2894	0	0	0	1	0	1	0
492	0	2657	0	0	0	1	0	0	0
432	216	1296	0	0	0	1	0	0	0
384	384	2064	0	0	0	0	0	0	0
858	0	1404	0	0	0	1	0	0	0
626	0	1392	0	0	0	1	0	0	0
1260	0	1134	0	0	0	1	0	0	1

Supporting information – S2 File

Raw data

1188	0	1426	0	0	0	1	0	0	1
810	0	864	0	0	0	1	0	0	0
216	0	1368	0	0	0	1	0	0	0
425	71	2761	0	0	0	1	0	0	0
382	0	1590	0	0	0	1	0	0	0
756	50	1411	0	0	0	1	0	0	0
115	115	1382	0	0	0	1	0	0	0
1728	0	4536	0	0	0	1	1	0	1
1075	0	2304	0	0	0	1	0	0	0
439	146	7027	0	0	0	1	1	0	0
3996	0	5106	1	0	0	1	1	1	1
810	0	2268	0	0	1	1	0	0	0
366	0	1391	0	0	0	1	0	0	0
798	798	5905	0	0	0	1	1	0	0
0	0	9102	1	0	1	1	1	0	0
1668	0	3503	1	0	0	1	0	0	1
677	0	1805	0	0	0	1	0	0	0
437	0	1638	0	0	0	1	0	0	0
2275	0	3413	1	0	1	1	0	0	1
1332	0	4262	0	0	0	1	0	0	1
1076	0	2070	0	0	0	1	0	0	0
346	0	3974	1	0	1	1	0	0	0
907	0	4385	0	0	0	1	1	0	0
NA	NA	NA	NA	NA	NA	NA	NA	NA	NA
176	0	1320	0	0	0	0	0	0	0
768	192	768	0	0	0	0	0	0	0
148	0	2820	0	0	0	0	0	0	0
461	230	1843	0	0	0	0	0	0	0
102	0	1840	0	0	0	0	0	0	0
407	0	1901	0	0	0	0	0	0	0
790	658	4869	0	0	0	0	1	0	0
843	0	1926	0	0	0	0	0	0	0
686	0	1372	0	0	0	0	0	0	0
672	252	3276	0	0	0	0	0	0	0
207	207	1865	0	0	0	0	0	0	0
297	74	2894	0	0	0	0	0	0	0
605	0	2923	0	0	0	0	0	0	0
647	277	2125	0	0	0	0	0	0	0

Raw data

421	60	1505	0	0	0	0	0	0	0
882	882	4116	0	0	0	0	0	0	0
693	139	1525	0	0	0	0	0	0	0
1109	0	2464	0	0	0	0	0	0	1
1001	0	1668	1	0	1	0	0	1	0
454	151	4082	0	0	0	0	0	0	0
229	229	1834	1	0	1	0	0	1	0
461	0	1382	0	0	0	0	0	0	0
176	353	1940	1	0	1	1	0	0	0

Supporting information – S2 File

Raw data

Basophilia
NA
0
0
0
0
0
0
0
0
1
0
1
0
1
0
0
0
0
0
0
0
0
0
0
0
0
0
0
0
0
0
0
0
0
0
0
0

Supporting information – S2 File

Raw data

0
0
0
0
0
0
0
0
0
0
0
0
0
1
0
0
0
0
0
0
0
0
0
NA
0
0
0
0
0
0
1
0
0
0
0
0
0
0

Supporting information – S2 File

Raw data

0
1
0
0
0
0
0
0
0
